# Development of Rapid and High-Precision Colorimetric Device for Organophosphorus Pesticide Detection Based on Microfluidic Mixer Chip

**DOI:** 10.3390/mi12030290

**Published:** 2021-03-09

**Authors:** Jiaqing Xie, Haoran Pang, Ruqian Sun, Tao Wang, Xiaoyu Meng, Zhikang Zhou

**Affiliations:** 1 College of Mechanical and Electronic Engineering, Northwest A&F University, Yangling 712100, China; xiejq@nwafu.edu.cn (J.X.); 18729071855@nwafu.edu.cn (H.P.); sunruqian@nwafu.edu.cn (R.S.); mxy917@nwafu.edu.cn (X.M.); zk_zhou@nwafu.edu.cn (Z.Z.); 2 Institute of Intelligent Manufacturing Technology, Shenzhen Polytechnic, Shenzhen 518055, China

**Keywords:** colorimetric device, organophosphorus pesticide residues, microfluidics, LCD mask photo-curing

## Abstract

The excessive pesticide residues in cereals, fruit and vegetables is a big threat to human health, and it is necessary to develop a portable, low-cost and high-precision pesticide residue detection scheme to replace the large-scale laboratory testing equipment for rapid detection of pesticide residues. In this study, a colorimetric device for rapid detection of organophosphorus pesticide residues with high precision based on a microfluidic mixer chip was proposed. The microchannel structure with high mixing efficiency was determined by fluid dynamics simulation, while the corresponding microfluidic mixer chip was designed. The microfluidic mixer chip was prepared by a self-developed liquid crystal display (LCD) mask photo-curing machine. The influence of printing parameters on the accuracy of the prepared chip was investigated. The light source with the optimal wavelength of the device was determined by absorption spectrum measurement, and the relationship between the liquid reservoir depth and detection limit was studied by experiments. The correspondence between pesticide concentration and induced voltage was derived. The minimum detection concentration of the device could reach 0.045 mg·L^−1^ and the average detection time was reduced to 60 s. The results provide a theoretical and experimental basis for portable and high-precision detection of pesticide residues.

## 1. Introduction

Organophosphorus pesticides are kinds of organic compound containing phosphorus, which is widely used in agricultural production, household health, garden management and other fields. Organophosphorus pesticides can effectively inhibit the activity of cholinesterase in the nervous system of animals and human beings, so that the acetylcholine decomposition process is suppressed, resulting in the accumulation of acetylcholine in nerve endings [1,2,3]. In recent years, the extensive use of organophosphorus pesticides has caused pollution in groundwater, surface water and soil, and its residues in food seriously threaten human health. The Codex Alimentarius Commission (CAC) has set strict standards of pesticide residues in food. For example, the maximum content of glufosinate (a typical organophosphorus pesticide) in most fruit and vegetables is 0.05 mg·L^−1^ [4,5,6]. At present, the detection methods of organophosphorus pesticide residues mainly include the precision instrumental analysis method and rapid detection method. The precision instrumental analysis method has superiority of high sensitivity and high selectivity, however, the shortcomings of a large volume, high cost, time-consuming duration and tedious pretreatment has limited the application of this method. Rapid detection methods mainly includes the biological detection method, immune method, enzyme inhibition method and biosensor method [7,8]. The enzyme inhibition method is based on the principle of colorimetry. The organophosphorus pesticides can inhibit the activity of acetylcholinesterase (AChE), slow down or stop the decomposition of acetylcholine. Therefore, acetylcholine, AChE and a chromogenic agent can be mixed to detect the sample concentration of pesticide residues. The presence of organophosphorus pesticides can be determined by the change of color or the change of physicochemical signal of enzyme reactions with a specific compound [9]. The rapid detection instrument and colorimetric instrument developed based on this principle has been able to realize the preliminary screening of pesticide residues in agricultural products. This method is currently facing many challenges, such as various accessories, inconvenience to carry, cumbersome operation, a lengthy process, poor accuracy, low sensitivity and repeatability. It is necessary to find a portable, low-cost and high-precision pesticide residue detection scheme to detect the concentration of organophosphorus pesticides.

Microfluidic chip technology integrates the processes of sample reaction, separation and detection involved in the fields of chemistry, biology and medicine into one chip, which makes the analysis equipment miniaturized and automated [10,11]. Jia [12] developed an impedance immunosensor based on a microfluidic chip for rapid detection of pesticide residues in vegetable samples. The microfluidic chip consisted of a detection microchamber inlet and outlet microchannel. A gold interdigitated array microelectrode (IDAM) was embedded in the microchannel of the microfluidic chip, which can be used for direct detection of practical samples. The research confirmed the value of microfluidic chips in pesticide detection, but the preparation of an immunosensor is difficult, and the detection time can be further compressed. Deng [13] developed a rapid semi-quantification detection method of trichlorfon residues by a microfluidic paper-based phosphorus-detection chip, the chip fabrication process was optimized. The author emphasizes the low cost of the chip, however, the durability of paper-based microfluidic chips was not investigated. Asghar [14] developed an innovative immuno-based microfluidic device that can rapidly detect and capture *Candida albicans* from phosphate-buffered saline (PBS) and human whole blood. The microchip technology showed an efficient capture of Candida albicans in PBS with an efficiency of 61–78% at various concentrations ranging from 10 to 10^5^ colony-forming units per milliliter (cfu∙mL^−1^). The mixing efficiency of microfluidic chip is also an important factor of pesticide detection device. Through reasonable structure design, the chip channel with high mixing efficiency at low Reynolds number can be obtained [15]. Fan [16] developed a rapid microfluidic mixer utilizing sharp corner structures, it can be potentially used in the fluid mixing in variety of lab-on-a-chip applications. The preparation method of the chip also needs to be considered. Plevniak [17] developed a low-cost, smartphone-based, 3D-printed microfluidic chip system for rapid diagnosis of anemia in 60 s, and a 3D-printed chip with a 5 gel lens on camera was assembled for capturing and analyzing color-scale results from the chip view-window. The color-scale image capture and analysis app written in-house was developed to extract RGB (red, green, and blue) peak values in the region of interest. The 3D printing technology greatly reduces the cost of microfluidic chip preparation, while the optical observation technology can achieve semi-quantitative blood sample observation. However, it is difficult to realize accurate quantitative analysis for samples with no significant color change. Previous studies have confirmed the advantages of microfluidic chips in the field of detection, especially in improving the mixing efficiency and reducing the consumption of reagents [18,19]. Meanwhile, the photoelectric detection method has higher detection accuracy and lower detection concentration than the image method.

Based on the above analysis, a microfluidic mixer chip which can realize efficient and quick mixing was developed. The chip was successfully fabricated by a self-development LCD mask photo-curing machine, and the corresponding colorimetric device was developed to realize the rapid and accurate detection of organophosphorus pesticides. Firstly, two kinds of mixing microchannel were designed, the mixing efficiency was evaluated by simulation, and the optimal microchannel structure was confirmed. Secondly, the microfluidic mixer chip was fabricated by a self-development LCD mask photo-curing machine, the influence of printing parameters on the microchannel accuracy was investigated. Finally, a portable colorimetric device was developed, the corresponding relationship between pesticide concentration and induced voltage was constructed, and quantitative detection of organophosphorus pesticides was realized. The results indicated that the detection efficiency was increased and the detection sample was reduced.

## 2. Experimental

### 2.1. Basic Theory

In order to achieve high-precision detection of organophosphorus pesticides, related theories involved in enzyme reaction, microfluidic chip design theory, mixing index analysis and laminar flow simulation were adopted. In addition, the calculation method of detection limit was developed to evaluate the performance of the colorimetric device. The theoretical contents related to the research are listed as follows.

**Figure 1:**
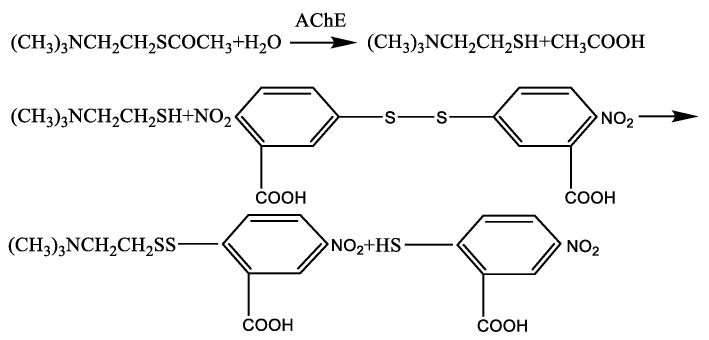
The principle of color reaction.

The color reaction was based on the Ellman method [20]. Under the catalysis of acetylcholinesterase (AChE), the thioacetylcholine iodide was hydrolyzed into thiocholine and acetic acid. The thiocholine reacted with 5, 5′-dithiobis (2-nitrobenzoic acid) to form a yellow product, as shown in Figure 1. The higher the AChE activity, the darker the color of the reagent after reaction. By contrast, AChE activity was inhibited and the color of reagent was lighter.

The diffusion coefficient *D* can be defined as [21],

(1) $$D=\frac{kT}{6\pi \mu r}$$

where *k* denotes the Boltzmann constant, *T* gives the absolute temperature, *μ* is dynamic viscosity and *r* is molecular radius. The diffusion coefficient is inversely proportional to the dynamic viscosity of the solution at a certain temperature.

The cross-section structure of the channel fabricated on the chip was rectangular, and the expression of Reynolds number can be expressed as follows,

(2) $$R_{e}=\frac{4\rho Av}{p\mu }$$

where A is the interface area, ρ is the liquid density, p is the wetting perimeter length, and v is the flow rate.

The relationship between transmitted light intensity *I* and incident light intensity $I_{0}$ can be expressed by the Lambert law,

(3) $$I=I_{0}10^{-\alpha \cdot l}$$

where l is the path length, α is the absorption coefficient describing the absorption capacity of a substance to light.

When the incident light is definite, the transmitted light is proportional to the concentration of the substance, and the above principle can be used to detect the concentration of pesticides.

The detection limit of the colorimetric device $x_{LOD}$ is determined by the following formula [22,23,24],

(4) $$x_{LOD}=\frac{2ts_{y}}{nt^{2}{s_{y}}^{2}-Ar^{2}}\times \left(ts_{y}\sum x_{i}-\sqrt{\frac{A^{2}r^{2}}{k}+Ar^{2}\sum {x_{i}}^{2}-n\frac{A}{k}}t^{2}{s_{y}}^{2}-At^{2}{s_{y}}^{2}\right)$$

where *t* is the Student’s *t*-function parameter, usually assumed to be *t* = 3 for convenience [22], $s_{y}$ is the standard deviation of the measurement, n is the tested number of concentrations, *k* designates the number of repeat measurements, *r* is the sensitivity as the slope of a linear fit, $x_{i}$ represents the pesticide concentration of the *i*-th experiment, the determinant in the denominator *A* is given by:

(5) $$A=n\sum {x_{i}}^{2}-{\left(\sum {x_{i}}^{2}\right)}^{2}$$

The Navier–Stokes equation [25] describing the behavior of incompressible fluid is used to simulate the mass and momentum transfer of fluid, which can be expressed as follows,

(6) $$\frac{\partial }{\partial x_{j}}\left(\rho u_{j}\right)=0$$

(7) $$\frac{\partial }{\partial x_{i}}\left(\rho u_{i}u_{j}\right)-\frac{\partial P}{\partial x_{i}}+\frac{\partial \tau_{ij}}{\partial x_{i}}$$

where ρ and $u_{j}$ are density and velocity vector, respectively, u is velocity vector of fluid, *P* is pressure on fluid, $\tau_{\mathrm{ij}}$ is stress tensor, respectively.

The mass flux is given by diffusion and convection, and the resulting mass balance is,

(8) $$\nabla \cdot \left(-D\nabla c+c\vec{u}\right)=0$$

where *c* gives the concentration.

In substances involved in color reactions, the AChE has the largest molecular size and the minimum diffusion rate, so the standard deviation of the AChE concentration on the cross-section at different positions of the microchannel was used to measure the uniformity of the fluid distribution, as well as the mixing efficiency of the chip [26].

### 2.2. Materials and Methods

The chromogenic agent (0.75 g·L^−1^) was prepared by mixing 5,5-dithio-bis-2-nitro benzoic acid (DTNB) (Shanghai Chemical Reagent Co., Ltd. Shanghai, China) with phosphoric acid buffer (0.1 mol·L^−1^). The concentration of AChE (Merck Life Science Technology Co., Ltd., Darmstadt, Germany) solution was 150 g·L^−1^. Different concentrations of glufosinate-ammonium (Lear Chemical Co., Ltd., Shenzhen, China) standard solutions were prepared to test the inhibition on AChE.

A transparent photosensitive resin (Shenzhen Novartis Intelligent Technology Co., Ltd., Shenzhen, China) was used to fabricate microfluidic mixer chip, the parameters of photosensitive resin after ultraviolet curing is shown in Table 1.

### 2.3. Characterization and Instruments

**Figure 2:**
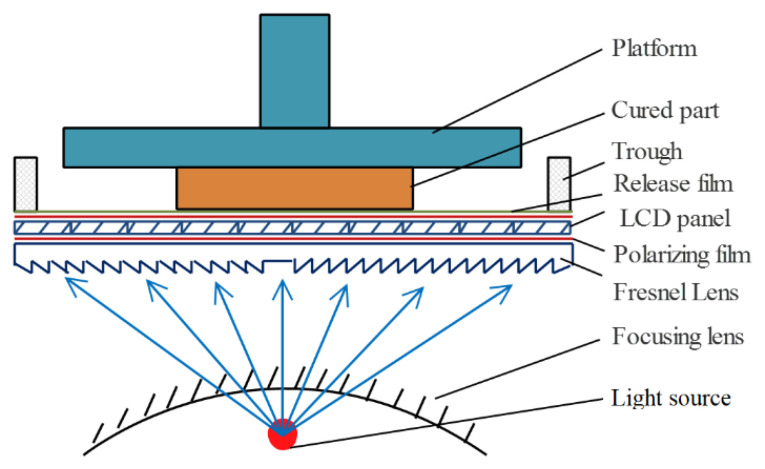
Schematic diagram of self-developed liquid crystal display (LCD) mask photo-curing machine.

A self-developed LCD mask photo-curing machine was adopted for microfluidic mixer chip fabrication. The device adopted 405 nm ultraviolet light as the curing light source and the LCD panel as the selective light transmission plate to realize the curing of photosensitive resin for complex parts. Figure 2 is the schematic diagram of the LCD mask photo-curing machine: the machine includes platform, Z axis, material trough and LCD panel, and the incident position of the light source can be controlled by the LCD panel [27]. The shape of cured resin parts was observed by the laser microscope (VHX-1000, KEYENCE Co., Ltd., Osaka, Japan) with a display resolution of height 0.005 μm and width 0.01 μm.

**Figure 3:**
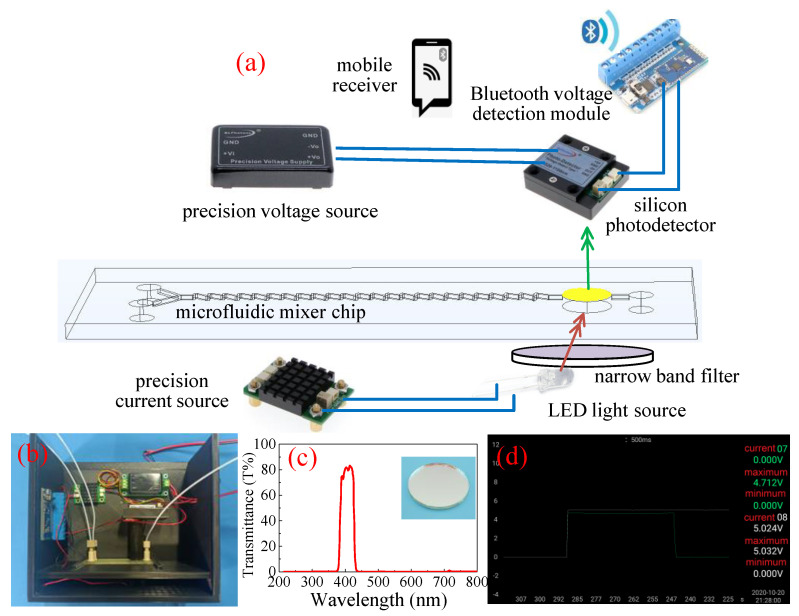
Schematic diagram of colorimetric device (**a**), colorimetric device after assembly (**b**), the absorption spectrogram of the narrow band filter (**c**), and self-developed mobile client (**d**).

The self-developed colorimetric device was adopted to detect the concentration of glufosinate-ammonium, a typical organophosphorus pesticide, the equipment included a light-emitting diode (LED) light source (Jinxin Photoelectric Technology Co., Ltd., Guangzhou, China), precision current source (Shenzhen wave particle Technology Co., Ltd., Shenzhen, China), precision voltage source (Shenzhen wave particle Technology Co., Ltd., Shenzhen, China), silicon photodetector (Shanghai Bose Intelligent Technology Co., Ltd., Shanghai, China) and Bluetooth voltage detection module. The schematic diagram of the equipment is shown in Figure 3a. Figure 3b is the colorimetric device after assembly: the LED light source is driven by a precision current source to ensure the stability of light intensity, and the customized narrow band filter with a diameter of 10 mm (Beijing Yongxing perception Instrument Co., Ltd., Beijing, China) was adopted to ensure that light of 385 nm to 430 nm wavelength could pass through, as shown in Figure 3c. The induced voltage of the silicon photodetector varies according to the intensity of the transmitted light, the induced voltage can be received by Bluetooth module and stored in the self-developed mobile client, and the resolution of silicon photodetector is 0.01 V, as shown in Figure 3d.

In the experiment, the two solvents were pushed into the microfluidic chip channel through the syringes driven by a micro injection pump (XFP02-B, Suzhou iFLYTEK Scientific Instrument Co., Ltd., Suzhou, China), the liquid flow rate was controlled by micro injection pump. The ultraviolet-visible spectrophotometer (UV-2250, Shimadzu Instrument Equipment Co., Ltd., Kyoto, Japan) was adopted to obtain the absorption spectra of the solution after reaction. The three-dimensional structure of the chip was designed by SOLIDWORKS (Dassault Systèmes SOLIDWORKS Corp, Massachusetts, USA) software. The multi-physics coupling analysis software COMSOL Multiphysics 5.5 (COMSOL Inc., Stockholm, Sweden) was applied to simulate the mixing process. The free quadrilateral mesh was adopted in the model, and the wall condition was no sliding, the fully developed flow was applied in the fluid inflow model, and the inlet pressure was 0. The MATLAB 2018 (Mathworks Inc., Natick, MA, USA) software was used to process the simulation images to obtain the concentration standard deviation on the chip channel section.

## 3. Results and Discussion

### 3.1. Influence of Microchannel Shape on Mixing Efficiency

**Figure 4:**
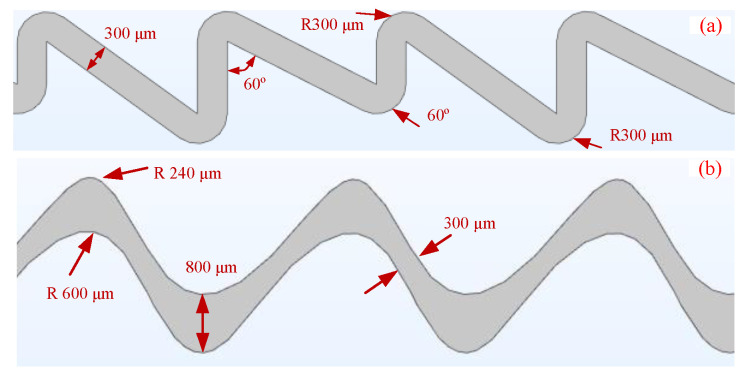
The designed two types of microstructures with curved channel (**a**), curved width variation channel (**b**).

Among the substances involved in the color reaction, the molecular size of AChE is the largest, while the diffusion coefficient is the smallest, so the purpose of microchip channel design is to realize the rapid and complete mixing of AChE. The length, width and height of AChE were 98 nm, 79 nm and 3 nm, respectively [28]. The physical parameters of the mixed liquid are shown in Table 2. The diffusion coefficient of AChE can be obtained by substituting the parameters into Equation (1), and the calculated result is 0.22 × 10^−11^ [29]. In addition, the flow rate of the microfluidic mixer chip should be controlled at a low speed to ensure complete reaction after mixing. Therefore, the aim of microfluidic chip design is to realize the efficient mixing of AChE at low Reynolds number (10–82). The main principle is to make the fluid stretch, compress, fold and surround in the channel to realize the rapid mixing of the liquid involved in the reaction, which can be achieved by changing the channel width and bending the channel shape. The change of the channel width makes the liquid flow perpendicular to the fluid flow direction, and the bending of the microchannel makes the pressure difference inside the liquid. In this case, the method of channel bending and width variation were proposed to improve the mixing efficiency [30,31]. Based on the above principles, two types of microchannel structure were designed, as shown in Figure 4. The channel volumes of the two types of structure are kept consistent to ensure the same mixing time.

**Figure 5:**
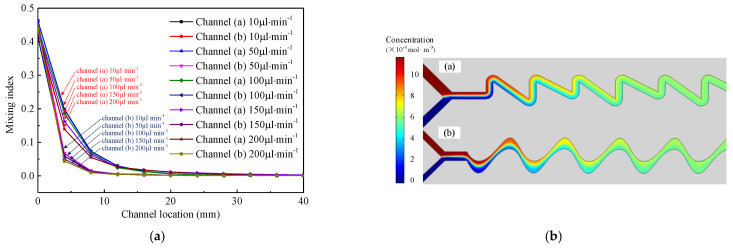
The simulation result of mixing index of two kinds of channels (**a**) and the simulated concentration distribution at the speed of 50 μL·min^−1^ (**b**).

The mixing process of the designed two kinds of microchannels were simulated, and the mixing index of AChE was compared, as shown in Figure 5a; the simulation results demonstrate that the mixing efficiency of channel structure (b) is significantly higher than that of channel structure (a). Meanwhile, the increase of channel flow has no significant effect on the improvement of mixing efficiency. By contrast, increasing the flow rate will reduce the mixing reaction time of the solution in the channel. Based on the above analysis, the flow rate of 50 μL·min^−1^ was selected in the experiment and the corresponding mixing time was 30 s; the simulated concentration distribution is shown in Figure 5b.

**Figure 6:**
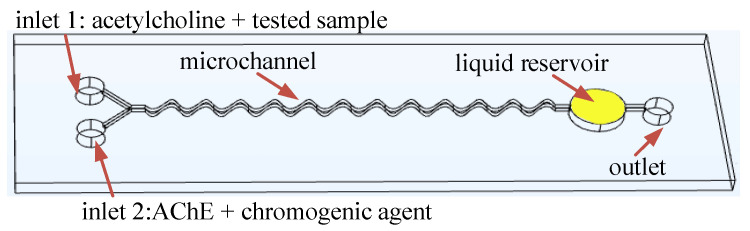
The diagram of the designed microfluidic mixer chip.

Based on the above analysis, a microfluidic mixer chip was designed, as shown in Figure 6, and contained two liquid inlets, a mixing channel, a liquid reservoir and an outlet. During the detection, the mixture of acetylcholine and the tested sample was injected into inlet 1, and AChE and chromogenic agent were injected into inlet 2.

### 3.2. Influence of Printing Parameters on Chip Fabrication Accuracy

**Figure 7:**
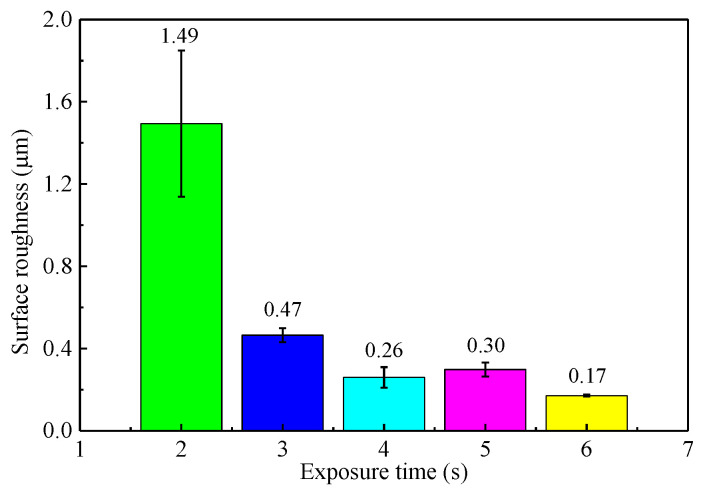
Relationship between ultraviolet (UV) exposure time and surface roughness.

The curing roughness and shape accuracy of the LCD mask photo-curing machine was investigated before preparing the microfluidic mixer chip. The relationship between UV exposure time and surface roughness of cured parts is shown in Figure 7. The suitable exposure time is between 2–6 s. When the exposure time is 6 s, the surface roughness decreases to the minimum of 0.17 μm.

**Figure 8:**
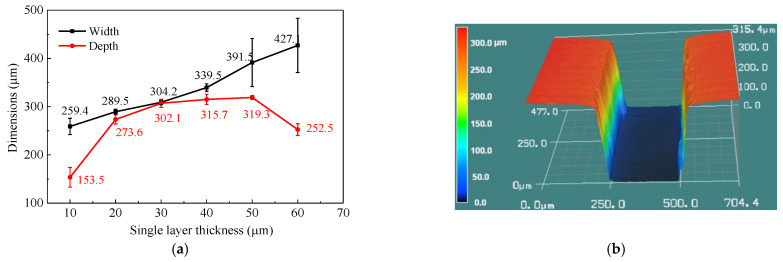
The influence of single layer curing thickness on channel size (**a**), and the cross-section shape of cured microchannel (**b**).

Due to the small size of the rectangular microgroove, there will certain deviation between the designed size and the actual preparation size during the curing process. In order to accurately prepare the rectangular microgroove with the target size, the influence of single layer printing thickness on channel size was investigated. A microchannel structure with a height and width of 300 μm was designed, the effect of printing layer thickness on dimensional accuracy was analyzed, and the exposure time is 6 s. The single layer curing thickness with minimum curing error appears at 30 μm, as shown in Figure 8a, the cross-section shape of the cured microchannel is shown in Figure 8b.

**Figure 9:**
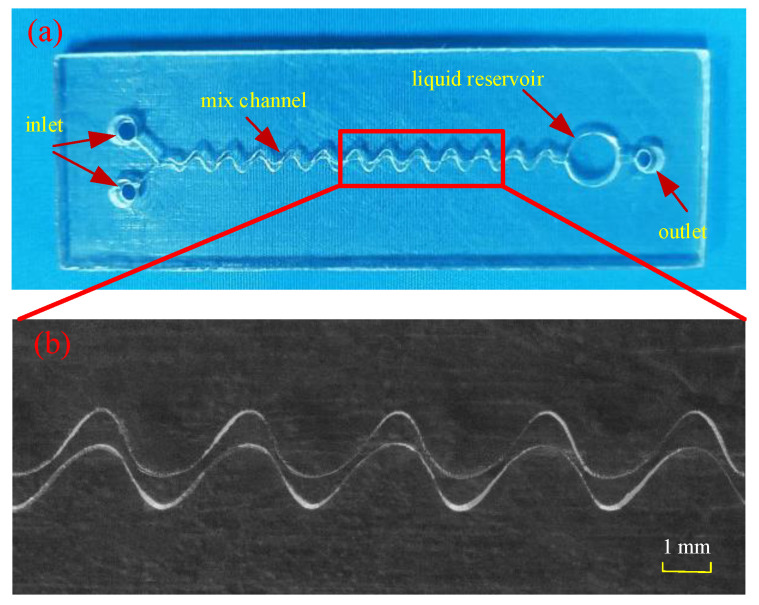
The microfluidic mixer chip fabricated by liquid crystal display (LCD) mask UV curing method: (**a**) overall structure and (**b**) scanning electron microscope (SEM) photograph of microchannel structure.

According to the above research, a rectangular cross-section microfluidic chip was prepared. The photograph of the fabricated microfluidic chip is shown in Figure 9a, The scanning electron microscope (SEM) photograph shows that the obtained structure has good size consistency, as shown in Figure 9b.

The chip was directly sealed with high transparency semi-solid acrylic material. The adhesive force between the semi-solid acrylic and the cured resin is large, and it will lose the bonding ability when contacting with water. In addition, the contact angle between acrylic and water is 57.5°, which is close to the cured resin (53.3°).

### 3.3. Relationshiop between Pesticide Concentration and Detection Voltage

**Figure 10:**
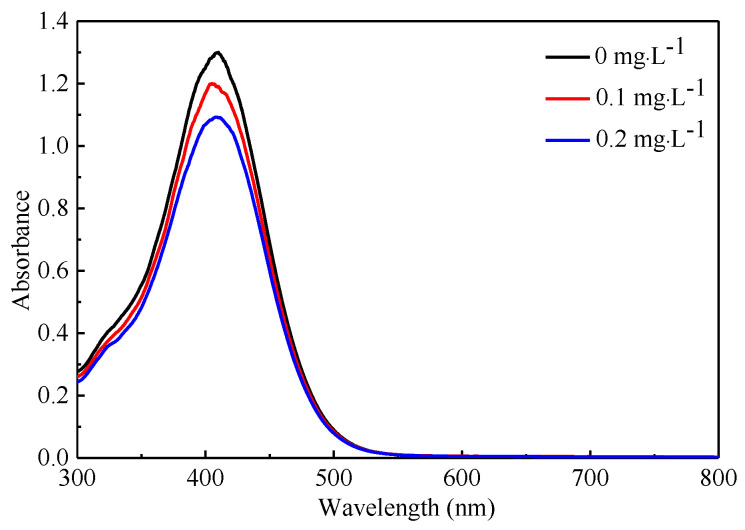
Absorption spectrum measurement results for 0, 1 and 2 mg∙L^−1^ concentrations.

The absorption spectrum measurement results for 0, 1 and 2 mg·L^−1^ concentrations of organophosphorus pesticide are shown in Figure 10. Theoretically, the sample without pesticide has the deepest color, corresponding to the highest light absorption value. In this test, whether the application of pesticide will change the absorption wavelength of the sample was observed. Therefore, pure water was selected as the contrast sample during the test. The experimental results indicated that the maximum absorption wavelength is 407 nm, it can be determined that the largest voltage difference would be obtained with 407 nm light source. In addition, the application of pesticides inhibited the color reaction and reduced the absorption peak.

**Figure 11:**
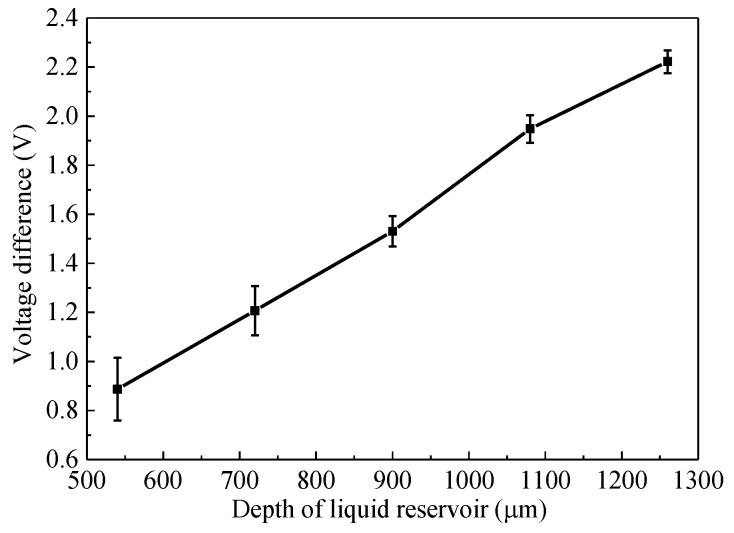
Influence of liquid reservoir depth on voltage difference.

The microfluidic chips with liquid reservoir depth of 540 μm, 720 μm, 900 μm, 1080 μm, and 1260 μm were separately prepared. The voltage difference between the pesticide concentration of 0 and 2 mg∙L^−1^. The influence of liquid reservoir depth on voltage difference is shown in Figure 11, the voltage difference increases linearly with the liquid reservoir depth, the test error decreases with the liquid reservoir depth.

**Figure 12:**
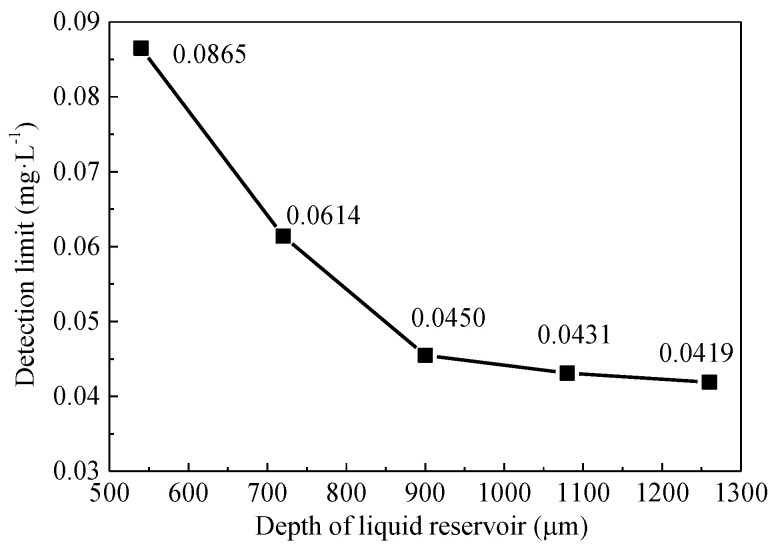
Relationship between liquid reservoir depth and detection limit.

Based on Equation (4), the detection limit of the device at different liquid reservoir depths was calculated, and each test was repeated 10 times; the test results are shown in Figure 12. The detection limit of the device decreases with the increasing of liquid reservoir depth. When the depth is greater than 900 μm, the decrease trend becomes slow. The reason is that the injection sample easily produces micro bubbles in the filling process when the liquid reservoir depth is too large, which affects the stability of the device. In addition, the injection time and the required sample volume increase with the liquid reservoir depth. After comprehensive analysis, the microfluidic chip with a liquid reservoir depth of 900 μm was selected as the mixing microfluidic chip of the device.

**Figure 13:**
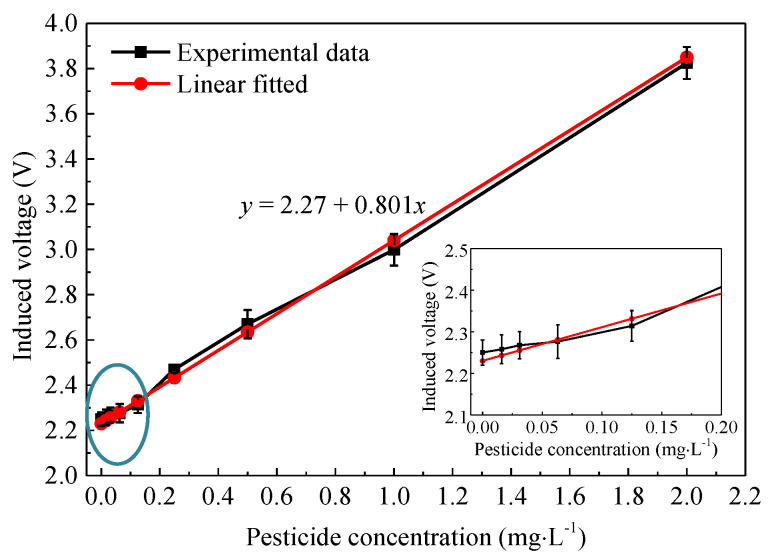
Relationship between induced voltage and pesticide concentration.

The relationship between induced voltage and pesticide concentration is shown in Figure 13. The test at each standard concentration was repeated 3 times, and the average values of each test were used for linear fitting. The fitted curve indicated that with the increase of glufosinate-ammonium content, the AChE activity was gradually suppressed, and the color reaction was inhibited, resulting in the increase of induced voltage. The linear relationship can be expressed as *y* = 2.27 + 0.801*x*, the linearity expression R^2^ can reach 0.985, while the detection limit is 0.045 mg·L^−1^.

**Figure 14:**
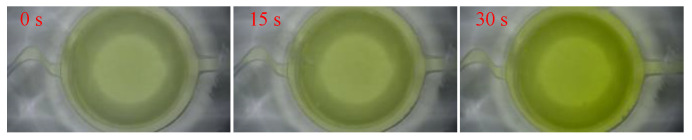
Color change of mixed liquid with time.

Figure 14 shows the color reaction process of the mixed liquid with time observed under microscope; after the reaction liquid mixed through the microchannel and reaches the liquid reservoir, it takes up to 30 s to achieve stable color reaction. The total test time is the sum of mixing time and reaction time, which can be reduced to 60 s.

Based on the above analysis, the accumulation time required for liquid mixing and reaction is 60 s at a flow rate of 50 μL·min^−1^ with a detection sample content of 25.2 μL. Compared with the existing pesticide residue detector, the detection time is reduced from more than 10 min to one minute, and the required sample content is very low. In addition, the detection limit (0.045 mg·L^−1^) is lower than the traditional pesticide detector (0.05 mg·L^−1^), which can meet the demand of pesticide residue detection with high precision [32,33]. However, all the experiments in this study were carried out at room temperature. In the follow-up study, an added temperature control function was proposed to further improve the device’s detection efficiency.

## 4. Conclusions

In this study, a colorimetric device for rapid detection of organophosphorus pesticide residues with high precision based on a microfluidic mixer chip was proposed. The detection system includes a microfluidic mixer chip and a silicon photodetector. The microfluidic simulation indicated that the microchannel with the characteristics of width variation and shape bending had better mixing efficiency. The optimal preparation parameters of the LCD mask photo-curing process were that the exposure time was 6 s and single layer thickness was 30 μm. The reaction liquid had the maximum absorption at 407 nm, and there was a linear relationship between pesticide concentration and induced voltage. The linearity expression R^2^ could reach 0.985, the minimum detection concentration of the system reached 0.045 mg·L^−1^ and the average detection time reduced to 60 s. The results provide a theoretical and experimental basis for portable and high-precision detection of pesticide residues.

## Figures and Tables

**Table 1:** Photosensitive resin parameters after ultraviolet curing.

Parameter	Value
Density (g·cm^−3^)	1.05–1.25
Tensile modulus (GPa)	1.8–2.8
Tensile strength (MPa)	64–72
Bending modulus (GPa)	1.8–2.3
Heat distortion temperature (℃)	44–47
Elongation at break (%)	8–13

**Table 2:** Physical parameters of mixed liquid.

Parameter	Value
Boltzmann constant (J·K^−1^)	1.38 × 10^−23^
Absolute temperature (K)	293.15
Dynamic viscosity (Pa·s)	10 × 10^−3^
Water density (Kg·m^−3^)	1000
